# Psychometric evaluation of the Adelphi Adherence Questionnaire (ADAQ©) in adults with osteoarthritis

**DOI:** 10.1186/s41687-024-00789-7

**Published:** 2024-10-14

**Authors:** Nathan Clarke, Andrew Trigg, Rob Arbuckle, Jan Stochl, Victoria Higgins, Sarah Bentley, James Piercy

**Affiliations:** 1 Adelphi Values, Adelphi Mill, Grimshaw Lane, Bollington, Macclesfield SK10 5JB UK; 2 Department of Kinanthropology, Charles University, Prague, Czechia; 3 Adelphi Real World, Bollington, UK

**Keywords:** Patient reported outcome development, PROs, Real-world evidence, Adherence, Validity

## Abstract

**Background:**

Medication non-adherence is a common issue in chronic illness. The World Health Organization has recognized a need for a valid and reliable method of measuring adherence to understand and mitigate non-adherence. This study aimed to psychometrically evaluate the English version of the Adelphi Adherence Questionnaire (ADAQ©), a questionnaire designed to assess patient-reported medication adherence across multiple therapy areas, in patients with Osteoarthritis (OA).

**Methodology:**

Data from the Adelphi OA Disease Specific Programme™, a survey of physicians and their consulting adult patients with OA conducted in the United States, November 2020 to March 2021, was used to assess the psychometric properties of the ADAQ. Patients completed the ADAQ, Adherence to Refills and Medication Scale (ARMS), Western Ontario and McMaster Universities Arthritis Index (WOMAC), and EQ-5D-3L. The measurement model of the 13-item ADAQ was assessed and refined using latent variable modelling (Multiple Indicator Multiple Cause, confirmatory and exploratory factor analyses, item response theory, Mokken scaling, and bifactor analyses). Correlational analyses (Spearman’s rank and polyserial as appropriate) with ARMS, WOMAC, and EQ-5D-3L scores assessed construct validity. Anchor- and distribution-based analyses were performed to estimate between-group clinically important differences (CID).

**Results:**

Overall, 723 patients were included in this analysis (54.5% female, 69.0% aged ≥ 60). Latent variable modelling indicated a unidimensional reflective model was appropriate, with a bifactor model confirming an 11-item essentially unidimensional score. Items 12 and 13 were excluded from scoring as they measured a different concept. The ADAQ had high internal reliability with omega hierarchical and Cronbach’s alpha coefficients of 0.89 and 0.97, respectively. Convergent validity was supported by moderate correlations with items of the ARMS, and physician-reported adherence and compliance. Mean differences in ADAQ score between high and low adherence groups yielded CID estimates between 0.49 and 1.05 points, with a correlation-weighted average of 0.81 points.

**Conclusion:**

This scoring model showed strong construct validity and internal consistency reliability when assessing medication adherence in OA. Future work should focus on confirming validity across a range of disease areas.

**Supplementary Information:**

The online version contains supplementary material available at 10.1186/s41687-024-00789-7.

## Introduction

Medication adherence is the degree to which medication is taken as prescribed [1]. Adherence differs from compliance in that it is an active process that presumes patient and physician agreement on recommendations rather than simply passively obeying advice [2]. Medication non-adherence is a common issue across chronic conditions (reported in up to 50% of patients) [3–6]. A report by the World Health Organization recognizes that a valid and reliable measure of adherence is required to better understand and mitigate medication non-adherence [7].

All methods of assessing medication adherence have their strengths and limitations. ‘Objective’ methods, such as counting remaining treatment doses, risk over-estimating adherence by not accounting for doses taken late or lost, they do not provide insight into reasons for non-adherence, and are costly to implement in practice [8]. Meanwhile, against regulatory standards, existing self-report measures such as the Adherence to Refills and Medications Scale (ARMS [9, 10]), Medication Adherence Rating Scale (MARS [11]) or the Morisky Medication Adherence Scale (MMAS [12]), to name a few, may not be considered fit to assess medication adherence behaviors and drivers across multiple conditions/medication administration methods. This is because they are often developed in a single disease area, a single country, without patient input, or in a single medication type; therefore, evidence for their wider content validity and reliability as well as relevant assessment concepts in certain therapy areas can be lacking [13]. The Adelphi Adherence Questionnaire (ADAQ©) was developed to address some of these gaps in content and evidence for existing measures.

The ADAQ was developed in accordance with professional and regulatory best practice for patient-reported outcome measures to measure adherence across a range of disease areas and diverse populations [14–18]. Measurement concepts were elicited from a review of qualitative literature and interviews with 57 adults with a wide range of health conditions (hypertension, asthma, multiple myeloma, psoriasis, diabetes, depression, multiple sclerosis, and/or schizophrenia) [19, 20]. These disease areas were chosen to ensure concepts accommodated an array of medication administration methods (e.g., topical, injected, inhaled), dosage regimes (e.g., daily, weekly), and patient demographics (e.g., different ages, cognitive abilities). Based on these measurement concepts and guidance from clinical experts, the ADAQ was developed and qualitatively evaluated among target populations to ensure respondent understanding and concept relevance [20–22]. While this research provided strong evidence of content validity, a scoring algorithm still needed developing and psychometric properties, such as reliability and construct validity, evaluating across different disease areas. This study focuses on psychometric evaluation of the ADAQ within an osteoarthritis (OA) setting, the first such psychometric evaluation to be performed (though psychometric evaluations in other health conditions are in progress). The psychometric properties of the ADAQ have not previously been evaluated in an OA population and OA patients were not included during qualitative research.

OA is a highly debilitating disease characterized by joint pain and movement difficulties. In addition to the pain and physical limitations experienced by the individual there is a wider burden on health systems and society; OA is estimated to cost the US approximately $136.8 billion annually in direct and indirect costs [23, 24]. To reduce the burden of OA it is essential that treatments effective at controlling pain and managing OA disability are developed and, importantly, that patients are adherent to prescribed long-term medication (such as non-steroidal anti-inflammatory drugs). Medication non-adherence has demonstrable associations with poorer health outcomes in OA patients [25].

The aim of this study was to assess the psychometric properties of the ADAQ, including the assessment of factorial structure (dimensionality), determination of a scoring algorithm, and evaluation of the validity and internal consistency reliability of the ADAQ in patients with OA based on analysis of real-world data.

## Materials and methods

### Data collection

This analysis was based on data from the Adelphi OA Disease Specific Programme (DSP™), a large cross-sectional, real-world, survey of patients with OA and their primary care physician, rheumatologist, or orthopedist. The DSP methodology has been previously published [26, 27], validated [28], and shown to be consistent over time [29]. Data were collected from November 2020 to March 2021. Patient and physician data were collected concurrently. This analysis included patients from the United States who were diagnosed with OA by their consulting physicians. Physicians were asked to complete a patient record form detailing patient demographics and clinical characteristics; these same patients were then asked to complete a patient self-complete form containing the ADAQ as well as the 12-item ARMS [9, 10], WOMAC [30], and EQ-5D-3L [31]. All patients who completed at least one item of the ADAQ were included in the analysis.

### Assessments

The ADAQ was developed as a 13-item patient-reported questionnaire designed to measure medication adherence [20–22]. A conceptual framework is provided in Table 1. Questions consisted of 4 items assessing medication adherence behaviors (e.g. missing a dose, taking less medication than prescribed), 8 items assessing drivers of medication adherence (e.g. forgetting, perceptions of treatment necessity), and one item assessing overall adherence to medication. Possible ordinal item responses for each question are shown in Supplementary Table 1. Item 9 asked about skipping taking medication or taking less due to cost and is designed for countries where patients pay out-of-pocket or have insurance (such as the United States) as opposed to more socialized healthcare systems (such as England); this item can be omitted if irrelevant in certain countries. An overall adherence summary score can be calculated based on the mean of the first 11 items and can range from 0 to 4, with lower scores indicating greater medication adherence. Overall scores are calculated if at least 8 items have been completed. This study developed and validated this scoring methodology in an OA population.

**Table 1:** Adelphi Adherence Questionnaire conceptual framework

		Item content
Treatment adherence	Medication adherence behaviors	1. Missing a dose
		2. Taking medication at a different time than prescribed
		3. Taking more medication than prescribed
		4. Taking less medication than prescribed
	Drivers of adherence	5. Forgetting to take medication
		6. Perceived treatment effectiveness
		7. Perceived treatment necessity
		8. Missing one or more dose due to being out of normal routine
		9. Missing one or more dose due to cost
		10. Missing one or more dose due to side effects
		11. Missing one or more dose due to social stigma
		12. Patient’s confidence they are taking medication as intended
		13. Overall adherence

**Fig. 1:**
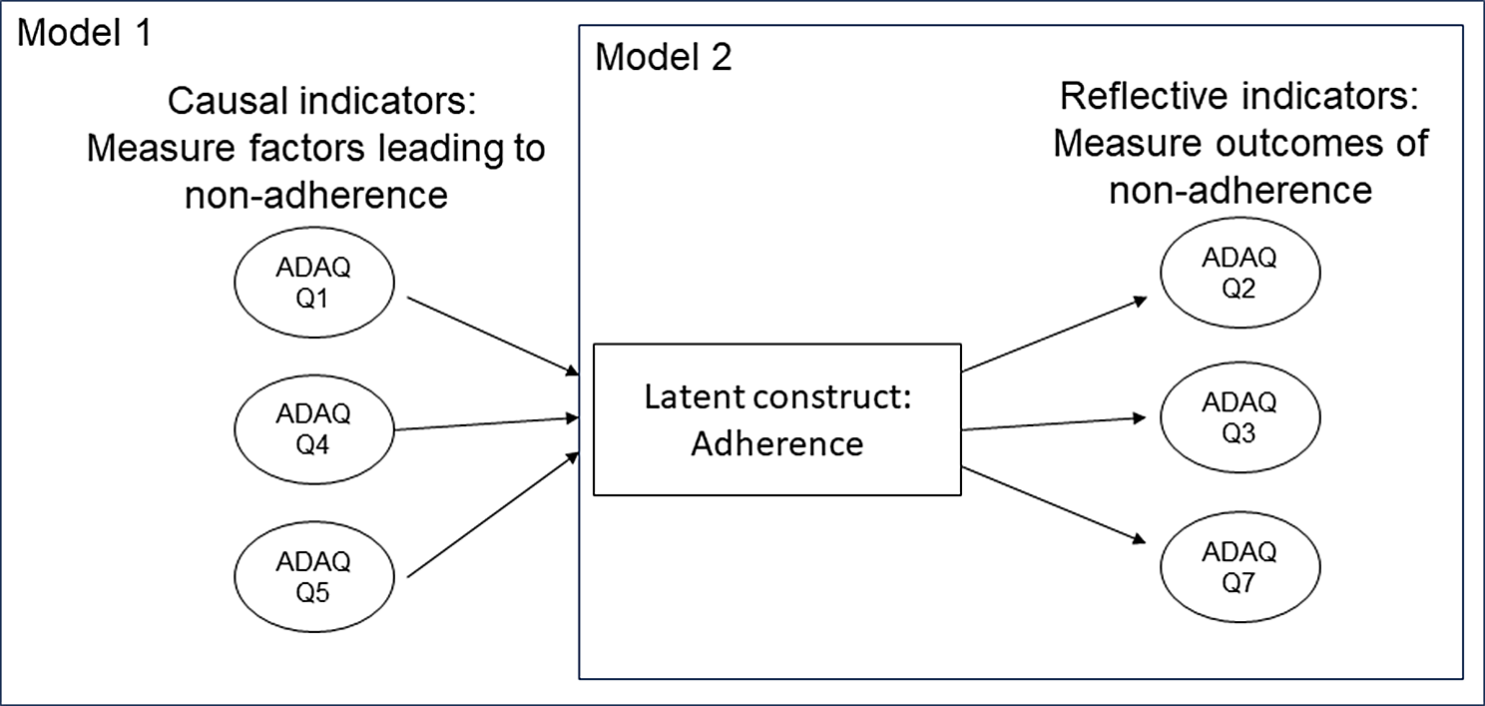
Illustrative causal/reflective indicator model (1) and fully reflective model for the Adelphi Adherence Questionnaire (2). *Notes* Not all questions are listed for concision, in the effect indicator model all questions would represent effects of non-adherence. *Abbreviations ADAQ* Adelphi Adherence Questionnaire

The ARMS is a 12-item patient-reported questionnaire assessing adherence. An overall sum score is calculated from all items and may range from 12 to 48, with lower scores indicating better adherence. Two subscales can also be calculated: ‘Taking medication as prescribed’, and ‘refilling medications on schedule’ [10].

The WOMAC is a 24-item patient-reported questionnaire developed for hip and knee OA [30]. It is divided into 3 subscales: Pain (5 items, range 0–20), stiffness (2 items, range 0–8), and physical functioning (17 items, range 0–68); higher scores indicate worse pain, stiffness, and physical functioning.

The EQ-5D-3L is a generic instrument for describing and valuing health in 5 dimensions: mobility, self-care, usual activities, pain/discomfort, and anxiety/depression [31]. Each dimension has 3 levels of response: no problems, moderate problems, and extreme problems.

A patient-reported single item measuring ‘satisfaction’ (‘Overall satisfaction with your medicine’) with 5 response categories (‘Extremely dissatisfied’ to ‘Extremely satisfied’), was administered, in addition to physician-completed single items measuring: ‘adherence’, ‘compliance’ and ‘satisfaction’. Details of responses can be found in Supplementary Table 1.

### Analyses

Two different measurement models underlying the ADAQ were evaluated. The first assumed a series of causal indicators (‘reasons for non-adherence’) gave rise to the latent construct of ‘adherence’, combined with several reflective indicators of adherence (‘extent of non-adherence’) [32–34]. The second potential model assumed all items were reflective indicators of adherence (illustrated in Fig. 1). The adequacy of these proposed measurement models was initially compared by fitting a multiple-indicator multiple-cause (MIMIC) model, and a unidimensional confirmatory factor analysis (CFA) model. The following global fit statistics for CFA models were calculated: standardized root mean square residual (SRMR; <0.10 acceptable), root mean square error of approximation (RMSEA; <0.1 acceptable), and comparative fit index (CFI; >0.95 acceptable) [35–37].

Assuming a fully reflective measurement model, various dimensionality analyses were also employed as recommended in the literature [38]. These analyses included evaluation of monotone homogeneity using a Mokken model—a non-parametric item response theory (IRT) model—and assessment of Loevinger’s scalability coefficient (where item clusters with coefficients > 0.5 reflect strong unidimensionality [39]), as well as exploratory graph analysis (EGA) using a glasso model with walktrap community detection algorithm [40–42].

Multidimensional structures suggested by any of these methods were subsequently explored through bifactor CFA modelling using a weighted least squares mean- and variance-adjusted (WLSMV) estimator. For the bifactor model, the proportions of explained common variance (ECV) across items by general and specific factors were then summarized (ECV > 0.80 is considered essential unidimensionality) [43]. Based on evaluation of measurement models and dimensionality, ADAQ scoring was then proposed.

Internal consistency of the ADAQ was assessed using McDonald’s omega coefficients and Cronbach’s alpha [44]. Construct validity of the ADAQ was assessed by comparing correlations to pre-defined hypotheses (which were determined based on consideration of the content of the questions for the different scores and whether measures were self- or physician-reported): ARMS total (*r* ≥ 0.50), ARMS taking medication as prescribed (*r* ≥ 0.30), ARMS refilling medications on schedule (*r* ≥ 0.30), physician-reported adherence (*r* ≥ 0.30), physician-reported compliance (*r* ≥ 0.30), physician-reported satisfaction (*r* ≥ 0.30), patient-reported satisfaction (*r* ≥ 0.30). Divergent hypotheses were also assessed: WOMAC scores (*r* ≤ 0.30) and EQ-5D-3L dimensions (*r* ≤ 0.30). To compare correlations between the ADAQ, patient-reported ARMS, and physician-reported adherence Steiger’s modification z using average correlations was utilized [45]. Response distributions were examined for each item to identify any unexpected or inappropriately skewed distributions.

Preliminary thresholds for between-group clinically important differences (CIDs) were estimated using anchor- and distribution-based methods [46, 47]. The anchor-based analysis results were prioritized for interpretation, with the distribution-based analyses considered to provide context for interpreting the anchor-based analysis. Anchors were formed using the ARMS total score, patient-reported satisfaction, and physician reported adherence, satisfaction, and compliance (details in Supplementary Table 2), where those with polyserial correlations with ADAQ score > 0.3 were retained. The difference in mean ADAQ score between the high and low adherence anchor groups were estimated for the calculation of CID, which were triangulated using a correlation-weighted average based on Fisher’s z transformation [48]. Supplementary distribution-based statistics comprised half a standard deviation (SD) and the standard error of measurement (SEM).

Measurement models, dimensionality, and correlation comparison analyses were performed in R (version 4.1.1) with the *psych* [49], *lavaan* [50], *mirt* [51], *MplusAutomation* [52], *BifactorIndicesCalculator* [53], *EGAnet* [54] and *mokken* [55], and *concor* [56] packages, and Mplus Version 8.1. All other analyses were performed in SAS Version 9.4.

## Results

Data from 723 patients formed the analysis population, of which 54.5% (*n* = 394) were female and 69.0% (*n* = 499) were aged 60 or over. Most patients were White (76.6%, *n* = 554), with 11.3% (*n* = 82) Black, 6.9% (*n* = 50) Hispanic/Latinx, and 5.2% (*n* = 37) other/non-white (Table 2). The joints most commonly affected by OA were knees (59.5%, *n* = 430), upper/middle/lower back (39.0%, *n* = 282), hips (27.0%, *n* = 195), and hands/fingers/thumbs (21.9%, *n* = 158). Clinicians rated 33.5% (*n* = 242) of patients as having mild OA, 49.4% (*n* = 357) moderate, and 16.6% (*n* = 120) severe; severity was not known or reported for 0.5% (*n* = 4) of patients. Physicians subjectively reported 42.2% (*n* = 257) of patients as completely adherent to medication (full breakdown in Table 2). Though the majority of treatments prescribed were orally administered (acetaminophen, ibruprofen, celecoxib, meloxicam, naproxen, naproxen/esomeprazole fixed dose combination, tramadol, codeine, morphine, hydrocodone, oxycodone, tapentadol, and the supplement chondroitin), there was some variability in treatment administration methods for diclofenac (*n* = 69, oral: 43, patch: 4, cream/topical: 22), hyaluronic acid (*n* = 23, oral: 4, subcutaneous/intramuscular injection: 4, other: 15),the supplement Glucosamine (*n* = 27, oral: 26, subcutaneous/ intramuscular injection: 1), intra-articular corticosteroid (*n* = 56, Oral:1, subcutaneous/ intramuscular injection: 25, other: 30), buprenorphine (*n* = 4, oral: 43, patch: 1, other: 3), and fentanyl (*n* = 4, patch/plaster: 4).

**Table 2:** Patient demographics

Characteristic	*n* = 723
Age, % (*n*)	
18–40	2.8 (20)
41–59	26.8 (194)
≥60 years	69.0 (499)
Missing	1.4 (10)
Female, % (*n*)	54.5 (394)
Ethnicity, % (*n*)	
White	76.6 (554)
Black	11.3 (82)
Hispanic/Latinx	6.9 (50)
Asian (Other)	2.1 (15)
Asian (Indian Subcontinent)	1.2 (9)
Mixed Race	0.7 (5)
South-East Asian	0.4 (3)
Middle Eastern	0.4 (3)
Native American	0.3 (2)
Employment status, % (*n*)	
Employed	41.4 (299)
Unemployed^a^	58.6 (424)
Education status, % (*n*)^b^	
High	25.6 (185)
Low	74.4 (538)
Insurance status, % (*n*)^c^	
High	43.8 (317)
Low	56.2 (406)
Physician-reported disease severity, % (*n*)	
Mild	33.5 (242)
Moderate	49.4 (357)
Severe	16.4 (120)
Don’t know	0.55 (4)
Physician-reported adherence, % (*n*)	*n* = 609
Completely adherent	42.2 (257)
Mostly adherent	41.9 (255)
Somewhat adherent	11.5 (70)
A little adherent	1.6 (10)
Not at all adherent	0.7 (4)
Too soon to tell	1.6 (10)
Don’t know	0.5 (3)

*Notes *^a^Unemployed includes: On long term sick leave, homemaker, student, retired, unemployed and don’t know

^b^Low education status is defined as having a high school diploma (or equivalent) or lower, high education status is defined as having above a high school diploma

^c^Low insurance status includes: Medicare, Medicaid, Medicare part D, Medicare medical savings account, Medicare advantage, Tricare/Veterans health care, unknown and none; High insurance status includes: Employer provided/sponsored insurance, partner/family member employer insurance, privately arranged insurance, health insurance exchange plan, Cobra, non-Medicare retired benefit and other

**Fig. 2:**
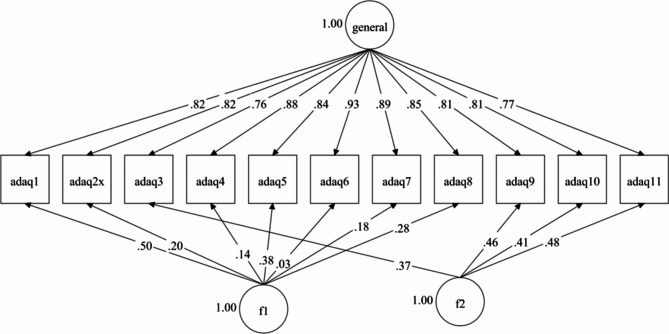
Bifactor model fitted to the 11 items that make up the Adelphi Adherence Questionnaire score. *Abbreviations ADAQ* Adelphi Adherence Questionnaire, *f* factor

**Fig. 3:**
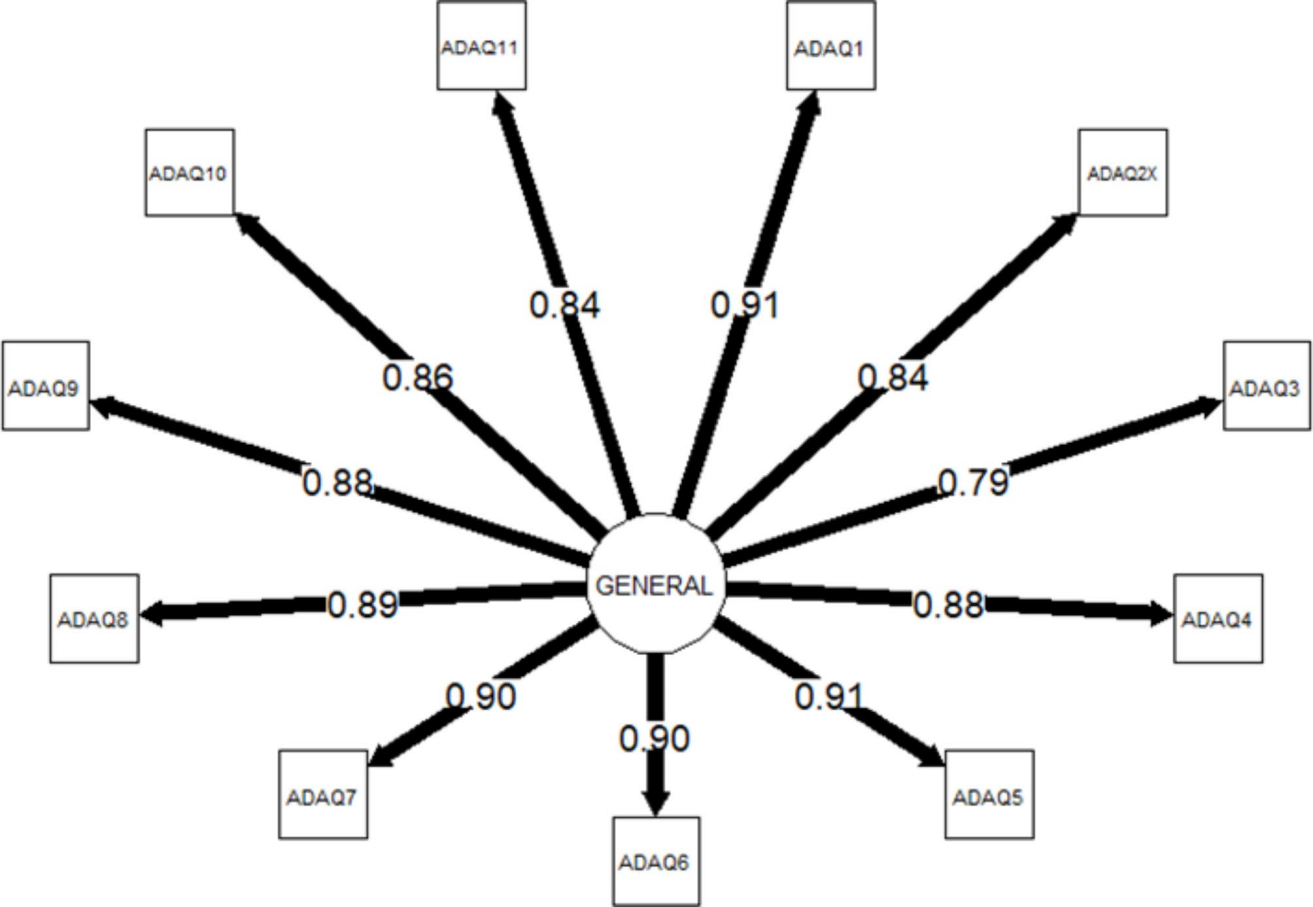
Unidimensional model fitted to the 11 items that make up the Adelphi Adherence Questionnaire score. *Abbreviations ADAQ* Adelphi Adherence Questionnaire

The fit of the MIMIC CFA model was generally poorer (SRMR = 0.1, CFI = 0.65, RMSEA = 0.12) in comparison to the unidimensional CFA model (SRMR = 0.05, CFI = 0.96, RMSEA = 0.16); therefore, a fully reflective measurement model was chosen for exploration of dimensionality. Inter-item correlations ranged from 0.41 to 0.89 (average 0.67), further supporting a fully reflective measurement model.

Mokken scale analysis demonstrated that items 1 to 11 clustered under a Loevinger’s scalability coefficient of 0.5, but items 12 (patient’s confidence in taking medication as intended) and 13 (patient perceived overall adherence) separated to form a separate cluster (See Supplementary Table 3). Bootstrapped EGA (500 iterations) suggested three factors: Items 1, 2, and 4–8 related to factor 1, items 3, and 9–11 to factor 2, and items 12 and 13 to factor 3 (see Supplementary Fig. 1). Unidimensional CFA model showed that factor loadings of items 12 and 13 ($\:{\lambda\:}_{12}=0.58$ and $\:{\lambda\:}_{13}=0.61$) were considerably lower compared to loadings of other items (ranging from 0.80 to 0.91). In summary, Mokken scale analysis, EGA, and CFA all suggested items 12 and 13 reflect a separate construct to the remaining 11 ADAQ items and were thus excluded from further analysis.

A bifactor model was fitted to the first 11 items of the ADAQ, where the use of two specific factors was guided by EGA. Figure 2 shows this model, which demonstrated acceptable fit across SRMR and CFI but not RMSEA (SRMR = 0.02, CFI = 0.99, RMSEA = 0.11) and a high ECV (0.85) for the general factor, supporting essential unidimensionality. This bifactor model and a unidimensional 11-item CFA (items 1 to 11; Fig. 3) were re-estimated using a full information maximum likelihood estimator which allowed direct comparison of both models with statistics penalizing for more complex models such as Akaike Information Criterion (AIC) or Bayesian Information Criterion (BIC). These statistics consistently favored the bifactor model (AIC: bifactor = 12201, unidimensional = 12616; BIC: bifactor = 12503, unidimensional = 12868; sample size adjusted BIC: bifactor = 12214, unidimensional = 12693).

Given the above, the ADAQ score was defined as the unweighted average of items 1 to 11. Items 12 and 13 can be summarized on their own for additional information, but do not contribute to the ADAQ score due to cumulative evidence that they may reflect a different construct to the other items. Item 13 relating to patient perception of adherence is also likely to violate local independence, a key assumption of CFA models. All subsequent analyses are based on the 11-item ADAQ score, which ranges from 0 to 4 where lower scores indicate greater adherence.

Convergent and divergent validity is summarized in Table 3. The majority of correlation size hypotheses were met, with the ADAQ showing strong convergent correlations with all measures of adherence and compliance, particularly the ARMS total score (0.774) and the ARMS taking medication as prescribed sub scale (0.798). The ADAQ showed a greater correlation with the patient-reported ARMS than physician-reported adherence (z = 11.2936, p-value < 0.001). ADAQ scores did not show convergent correlation with either patient- or physician-reported satisfaction.

**Table 3:** Correlational analysis of the Adelphi Adherence Questionnaire score with other patient- and physician-reported measures

Score	Hypothesized correlation	Estimated correlation	Hypothesis met?*
ARMS total	≥0.5	0.774 (S)	Yes
ARMS taking medication as prescribed	≥0.3	0.798 (S)	Yes
ARMS refilling medications on schedule	≥0.3	0.382 (S)	Yes
Physician-reported adherence	≥0.3	0.448 (P)	Yes
Physician-reported compliance	≥0.3	−0.345 (P)	Yes
Physician-reported satisfaction	≥0.3	0.180 (P)	No
Patient-reported satisfaction	≥0.3	−0.298 (P)	No
WOMAC pain	≤0.3	0.207 (S)	Yes
WOMAC stiffness	≤0.3	0.200 (S)	Yes
WOMAC physical functioning	≤0.3	−0.123 (S)	Yes
EQ-5D-3L mobility	≤0.3	0.013 (P)	Yes
EQ-5D-3L self care	≤0.3	0.267 (P)	Yes
EQ-5D-3L usual activities	≤0.3	0.194 (P)	Yes
EQ-5D-3L pain	≤0.3	0.160 (P)	Yes
EQ-5D-3L anxiety/depression	≤0.3	0.185 (P)	Yes

*Abbreviations ARMS* Adherence to Refills and Medication Scale, *WOMAC* Western Ontario and McMaster Universities Arthritis Index, *S* spearman’s rank, *P* polyserial

*Estimated correlation higher than the hypothesized threshold [69]

The internal consistency reliability coefficients omega hierarchical and Cronbach’s alpha were 0.89 and 0.97, respectively. Cronbach’s alpha did not increase or notably decrease after deletion of any item from the final 11-item version of the ADAQ, showing that all contributed towards reliability and supporting retention of all items. All anchor measures for score interpretation analyses were sufficiently correlated except for physician-reported satisfaction (*r* = 0.263) and compliance (*r* = 0.247). Mean differences in ADAQ score between high and low adherence groups yielded CID estimates between 0.49 and 1.05 points, with a correlation-weighted average of 0.81 points (Table 4). Distribution-based estimates were 0.40 (half SD) and 0.20 (SEM).

**Table 4:** Preliminary clinically important difference estimates based on anchor measures

Anchor	Correlation	CID estimate (95% CIs)
ARMS total score	0.650	0.78 (0.69–0.88)
Patient-reported satisfaction	0.300	0.49 (0.32–0.67)
Physician-reported adherence	0.444	1.05 (0.73–1.36)
**Correlation-weighted average** ^**a**^		**0.81 (0.57–1.04)**

*Notes *^a^Correlations transformed using Fisher’s z transformation

## Discussion

Latent variable modelling was used to evaluate the ADAQ measurement model. This included factor analysis (featuring unidimensional CFA, MIMIC, and bifactor models), EGA, and Mokken scaling. MIMIC, bifactor, and CFA models are subtypes of structural equation measurement models. MIMIC models were used to test the initially proposed conceptual structure of the ADAQ (i.e., that it contains both drivers, and reflections of adherence). EGA is similar to exploratory factor analysis and was used to evaluate the number of factors underlying the ADAQ following unacceptable fit of initially proposed MIMIC models. Mokken scaling (related to item response theory models, but with non-parametric assumptions) was used to help identify potential scales for a unidimensional ADAQ score. Both EGA and Mokken scaling supported the removal of items 12 and 13 from an ADAQ total score, with the latter suggesting the appropriateness of an 11-item unidimensional “adherence” score. CFA and bifactor models were used to evaluate the fit of an 11-item ADAQ score and further assess whether it was appropriate to consider an “essentially unidimensional” structure for the ADAQ (i.e. do all items reflect the overarching concept “adherence”). An essentially unidimensional structure was supported, with a bifactor model (featuring a strong general factor and 2 specific factors providing the best fit), particularly with regard to SRMR, which has been demonstrated to be more appropriate for use in categorical/ordinal factor analyses than RMSEA [37].

The results from the Mokken scale analysis and bifactor modelling supported an essentially unidimensional measurement model for the ADAQ, where the unweighted average of items 1 to 11 of the ADAQ represents the overall ADAQ score. We note that the utility of item weighting of total scores remains a topic of ongoing debate in the methodological literature but was also chosen based on practical considerations (i.e., to facilitate its application in broad research settings, and to multiple disease areas) [57–62]. Items 12 and 13 provided additional information but did not contribute to ADAQ scoring because latent variable modelling suggested they represent a different construct. Item 12 assessed the patient’s confidence that they are taking their medication correctly and item 13 provided a patient’s perception of their own adherence. As such, while these items provide useful context to the more in-depth assessment of adherence behaviors provided by items 1–11, these items can still be administered but should be scored separately. Possible scores for the summary score range from 0 to 4 where 0 is full adherence and 4 is complete non-adherence. Preliminary CID estimates indicated that a change in ADAQ score of 0.81 represents a clinical important difference in adherence in the context of OA.

ADAQ scoring showed strong internal consistency reliability with omega hierarchical and Cronbach’s alpha coefficients of 0.89 and 0.97, respectively.

ADAQ scores showed strong convergent correlation with ARMS scores and physician-reported compliance and adherence, and divergent correlation with the WOMAC and EQ-5D-3L scores. Perhaps unsurprisingly, the only areas in which ADAQ scoring did not achieve hypothesized correlation thresholds were with physician- and patient-reported satisfaction. In CFA analysis we found the model that best fit the ADAQ was a unidimensional reflective model meaning ADAQ scores likely represent the effects of non-adherence rather than the causes of non-adherence. It is likely that patient dissatisfaction is a cause of non-adherence rather than an effect of non-adherence [63]. In addition, while one may expect satisfaction to correlate with adherence, there are likely many other factors involved in patient satisfaction [64]. Similarly, there are likely factors other than treatment satisfaction impacting adherence such as the cost of treatment and medication side-effects. The physician-reported satisfaction question particularly focused on level of pain control, which is just one aspect of non-adherence captured by the ADAQ. The ADAQ provides a tool with which physicians can better understand the myriad factors associated with medication adherence.

The fact that the ADAQ showed higher convergent correlation with the patient-reported ARMS than physician-reported medication adherence (tested using Steiger’s modification z using average correlations [45]), highlights one of the key strengths of the ADAQ as a patient-reported measure of adherence. It has been shown in other disease areas that there is often discordance between physician- and patient-reported adherence in chronic conditions [65]. Physicians can only generally report adherence based on clinical test results, symptom assessment, information collected from patients and how frequently patients are collecting their prescriptions, and therefore may not be aware of patients missing doses, losing doses, or not taking medication on time. Clinical measures of adherence also lack the context surrounding non-adherence. The fact that the ADAQ correlates better with patient-reported measures indicates that it is reliably and accurately able to capture the nuance of patient-reported adherence that may be lacking from assessments based on physician judgment.

Response distribution issues could potentially limit ability to distinguish between some patients [66, 67]. Ceiling effects are particularly common among self-report measures of adherence due to social desirability and memory biases [66]. The scoring algorithm here led to nearly 20% of patients scoring 0 on medication adherence potentially indicating a ceiling effect. It has been shown, however, that patients with more severe OA are more likely to be adherent to their medication than those with mild OA [68]. Over half of our cohort were considered moderate to severe by their physician which could explain the high percentage of patients scoring 0 for adherence.

This study has several limitations. In terms of methodology, the cross-sectional nature of DSPs means we were unable to study the test-retest reliability of the ADAQ and ability to detect change in adherence, these properties should therefore be addressed in future research. The very high internal consistency coefficients observed could be argued to suggest a degree of redundancy. However, given that all ADAQ items are focused on different adherence behaviors and drivers, it was judged that all items were of value to retain from a content validity perspective. Nevertheless, further item reduction or creation of a short-form could be considered in the future. Given the ADAQ asks patients to report adherence retrospectively, there is the potential for recall bias particularly in patients on medications taken less frequently. Similarly, no recall period was set for the ADAQ to ensure its utility across a wide range of disease and treatment areas with differing dosing schedules (for example, if a 7-day recall period was included, this would not be appropriate for a patient whose treatment is a monthly injection). This does however mean patients could be reporting adherence over differing time periods. Further, as noted, the aim is that the ADAQ should be used across disease areas and treatment modalities including a range of types of treatments and supplements. As such, further validation in other disease populations with a broader range of treatments/supplements is also recommended, to confirm if the measurement properties are consistent across populations. It should also be acknowledged that it could be debated whether considering ‘adherence’ is relevant/appropriate for treatments such as physician-administered intra-articular injections; however, even those require the patient to attend appointments, and so it could be considered a relevant consideration. Moreover, in this study, most patients receiving such treatment were also taking treatments that were self-administered. Despite these limitations our analysis allowed us to develop a robust scoring system in a real-world setting to effectively measure adherence in consulting patients with OA. Data analyzed in this study were collected between November 2020 and March 2021; thus during the COVID-19 pandemic. While these authors see no reason to expect the instrument measurement properties would be different for this reason, it is possible that patients may have been more (perhaps due to simpler routines, less distractions) or less (perhaps due to less contact with healthcare professionals) adherent at that time. Confirmation of the findings presented here in data collected following the pandemic would provide insight/reassurance around this point.

## Conclusions

This paper outlines results of a psychometric evaluation of the ADAQ in a large and diverse OA patient population. The analysis demonstrated that the 13-item ADAQ and 11-item scoring algorithm developed herein have strong construct validity and internal consistency reliability when measuring medication adherence in the adult OA population. This version of the ADAQ provides a valid measure of patient-reported adherence to medication and addresses an unmet need for a generic medication adherence measure for use across a variety of conditions in real-world studies and routine clinical practice. To support the use of the ADAQ in the assessment of patient-reported adherence, future work should focus on establishing interpretation guidelines for the ADAQ score and test-retest reliability as well as its validity in other disease areas and confirmation of cross-cultural validity.

## Electronic supplementary material

Below is the link to the electronic supplementary material.

Supplementary Material 1

## Data Availability

All data that support the findings of this study are the intellectual property of Adelphi Real World. All requests for access should be addressed directly to Nathan Clarke at nathan.clarke@adelphivalues.com.

## Abbreviations

ADAQ©: Adelphi Adherence Questionnaire
AIC: Akaike Information Criterion
ARMS: Adherence to Refills and Medications Scale
BIC: Bayesian Information Criterion
CFA: Confirmatory Factor Analysis
CFI: Comparative Fit Index
CIDs: Clinically Important Differences
DSP: Disease Specific Programme
ECV: Explained Common Variance
EGA: Exploratory Graph Analysis
IRT: Item Response Theory
MARS: Medication Adherence Rating Scale
MIMIC: Multiple-indicator Multiple-cause
MMAS: Morisky Medication Adherence Scale
OA: Osteoarthritis
PRO: Patient Reported Outcome
RMSEA: Root Mean Square Error of Approximation
SD: Standard Deviation
SEM: Standard Error of Mean
SRMR: Standardized Root Mean Square Residual
WLSMV: Weighted Least Squares Mean- and Variance-adjusted Estimator
WOMAC: Western Ontario and McMaster Universities Arthritis Index
