# Supplementary material for: Psychometric evaluation of the Adelphi Adherence Questionnaire (ADAQ©) in adults with osteoarthritis
Source: J Patient Rep Outcomes. 2024 Oct 14;8:118. doi: 10.1186/s41687-024-00789-7 (PMC11473480; doi:10.1186/s41687-024-00789-7)
Supplement: Supplementary file 1 — Supplementary Material 1 [file 41687_2024_789_MOESM1_ESM.pdf]

Supplementary Appendix

Supplementary Table 1: Table showing possible responses to questions of the Adelphi Adherence Questionnaire

**Question number**	**Possible answers**
**1**	Never, rarely, sometimes, often, I stopped taking my medication completely
**2**	Never, rarely, sometimes, often, I stopped taking my medication completely, I have not been advised to take my medication(s) at a certain time
**3**	Never, rarely, sometimes, often, I stopped taking my medication completely
**4**	Never, rarely, sometimes, often, I stopped taking my medication completely
**5**	Never, rarely, sometimes, often, I stopped taking my medication completely
**6**	Never, rarely, sometimes, often, I stopped taking my medication completely
**7**	Never, rarely, sometimes, often, I stopped taking my medication completely
**8**	Never, rarely, sometimes, often, I stopped taking my medication completely
**9**	Never, rarely, sometimes, often, I stopped taking my medication completely
**10**	Never, rarely, sometimes, often, I stopped taking my medication completely
**11**	Never, rarely, sometimes, often, I stopped taking my medication completely
**12**	Not at all confident, a little confident, confident, very confident, I stopped taking my medication(s) completely
**13**	None of the time, a little of the time, some of the time, most of the time, all of the time*

Notes: *Responses are reverse coded

Supplementary Table 2: Anchors and their defined scales used to estimate clinically important differences

**Anchor #**	**Anchor Measure**	**Definition**
**1**	ARMS Total Scored	• High adherence (8-15)<br>• Low adherence (16-48)

**2**	Patient-reported<br>Satisfaction with current treatment	• High adherence (Moderately/Completely satisfied)<br>• Low adherence (Moderately/Completely dissatisfied)
**3**	Clinician-reported Adherent to current treatment regimen	• High adherence (Completely/Mostly adherent)<br>• Low adherence (A little/Not at all adherent)
**4**	Clinician-reported Satisfaction with current treatment	• High adherence (Moderately/Completely satisfied)<br>• Low adherence (Moderately/Completely dissatisfied)
**5**	Clinician-reported Compliance with current treatment regimen	• High adherence (Fully/Fairly compliant)<br>• Low adherence (Poor compliance/Not at all)

Abbreviations: Adherence to Refills and Medication Scale, ARMS

Supplementary Table 3: Scales as per increasing level of Loevinger's scalability coefficient (Hi). Numbers indicate item clusters forming monotone homogeneity Mokken model under corresponding Hi.

	0.3	0.35	0.4	0.45	0.5	0.55	0.6
**ADAQ1**	1	1	1	1	1	1	1
**ADAQ2**	1	1	1	1	1	1	1
**ADAQ3**	1	1	1	1	1	1	1
**ADAQ4**	1	1	1	1	1	1	1
**ADAQ5**	1	1	1	1	1	1	1
**ADAQ6**	1	1	1	1	1	1	1
**ADAQ7**	1	1	1	1	1	1	1
**ADAQ8**	1	1	1	1	1	1	1
**ADAQ9**	1	1	1	1	1	1	1
**ADAQ10**	1	1	1	1	1	1	1
**ADAQ11**	1	1	1	1	1	1	1
**ADAQ12**	1	1	1	1	2	2	0
**ADAQ13**	1	1	1	1	2	2	0

Abbreviations: Adelphi adherence questionnaire, ADAQ

Confidential - Not for Public Consumption or Distribution

Supplementary Figure 1: Bootstrapped exploratory graph analysis item clustering

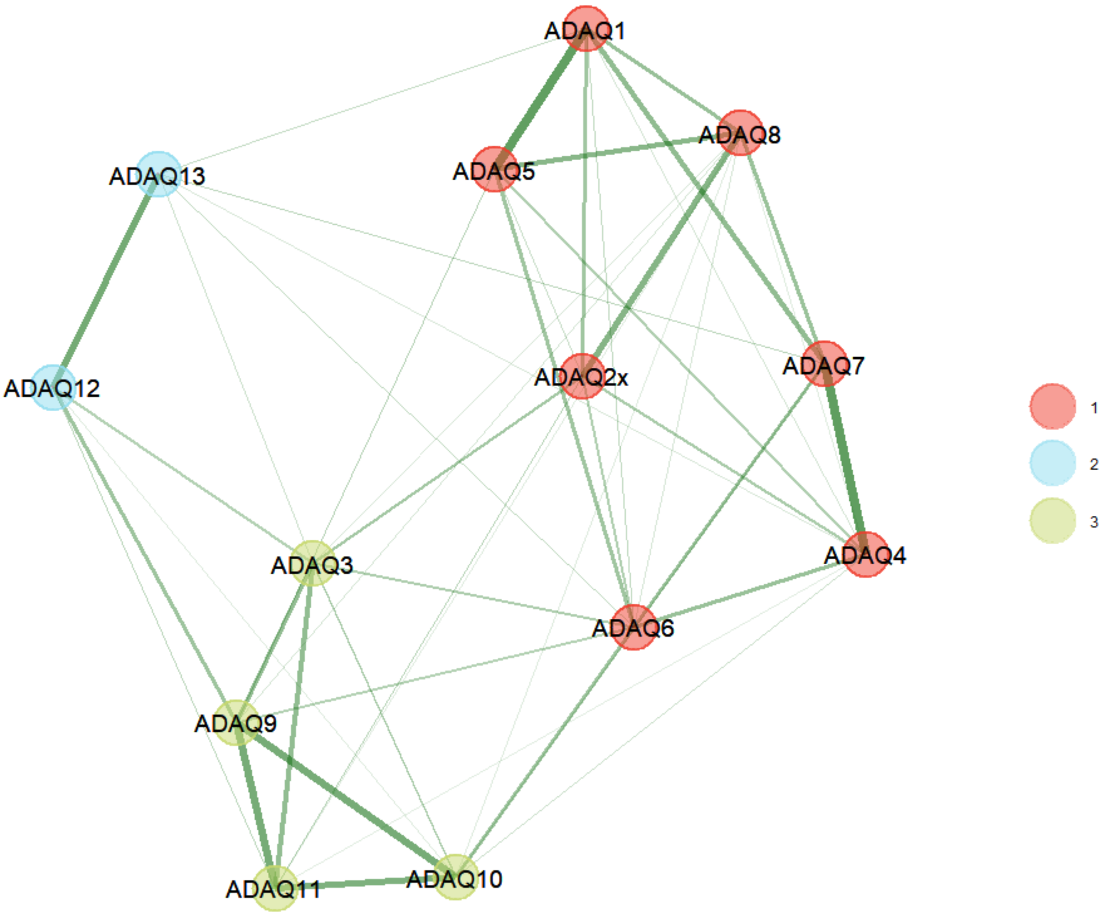

Abbreviations: Adelphi adherence questionnaire, ADAQ
